# Supplementary material for: Excess reciprocity distorts reputation in online social networks
Source: Sci Rep. 2017 Jun 14;7:3551. doi: 10.1038/s41598-017-03481-7 (PMC5471239; doi:10.1038/s41598-017-03481-7)
Supplement: Supplementary file 1 — Supplementary Information [file 41598_2017_3481_MOESM1_ESM.pdf]

Supplementary information for:

Excess reciprocity distorts reputation in online social networks

Giacomo Livan,[1,2] Fabio Caccioli,[1,2] and Tomaso Aste[1,2]

[1] *Department of Computer Science, University College London, Gower Street, London WC1E 6EA, UK*
[2] *Systemic Risk Centre, London School of Economics and Political Sciences, Houghton Street, London WC2A 2AE, UK*

S.1. DISTRIBUTIONS OF RATINGS AND REPUTATIONS

As it is often the case in networked systems (see, e.g., [1]), links are not distributed homogeneously between the nodes of the three platforms we analyse. Fig. S.1 shows the distributions of the four quantities we introduce in Eqs. (1) and (2) of the main paper, i.e. the number of reciprocated positive ratings (γ^+), reciprocated negative ratings (γ^-), unreciprocated positive ratings (ϕ^+), and unreciprocated negative ratings (ϕ^-) received by each user in the platform. Upon aggregation, these four quantities amount to the number of ratings received by each user (i.e. $\gamma^+ + \gamma^- + \phi^+ + \phi^-$), whose empirical distribution is shown in the left panel of Fig. S.2 together with the empirical distribution of the number of ratings given by each user to other peers in the platform. As it can be seen, all of these distributions are heavy tailed, and in Fig. S.2 we also report a calibrated probability distribution function of the form $p(x) \sim x^{-\alpha}$. In all cases we obtain values such that $2 < \alpha < 4$ (see the caption in Fig. S.2 for further details).

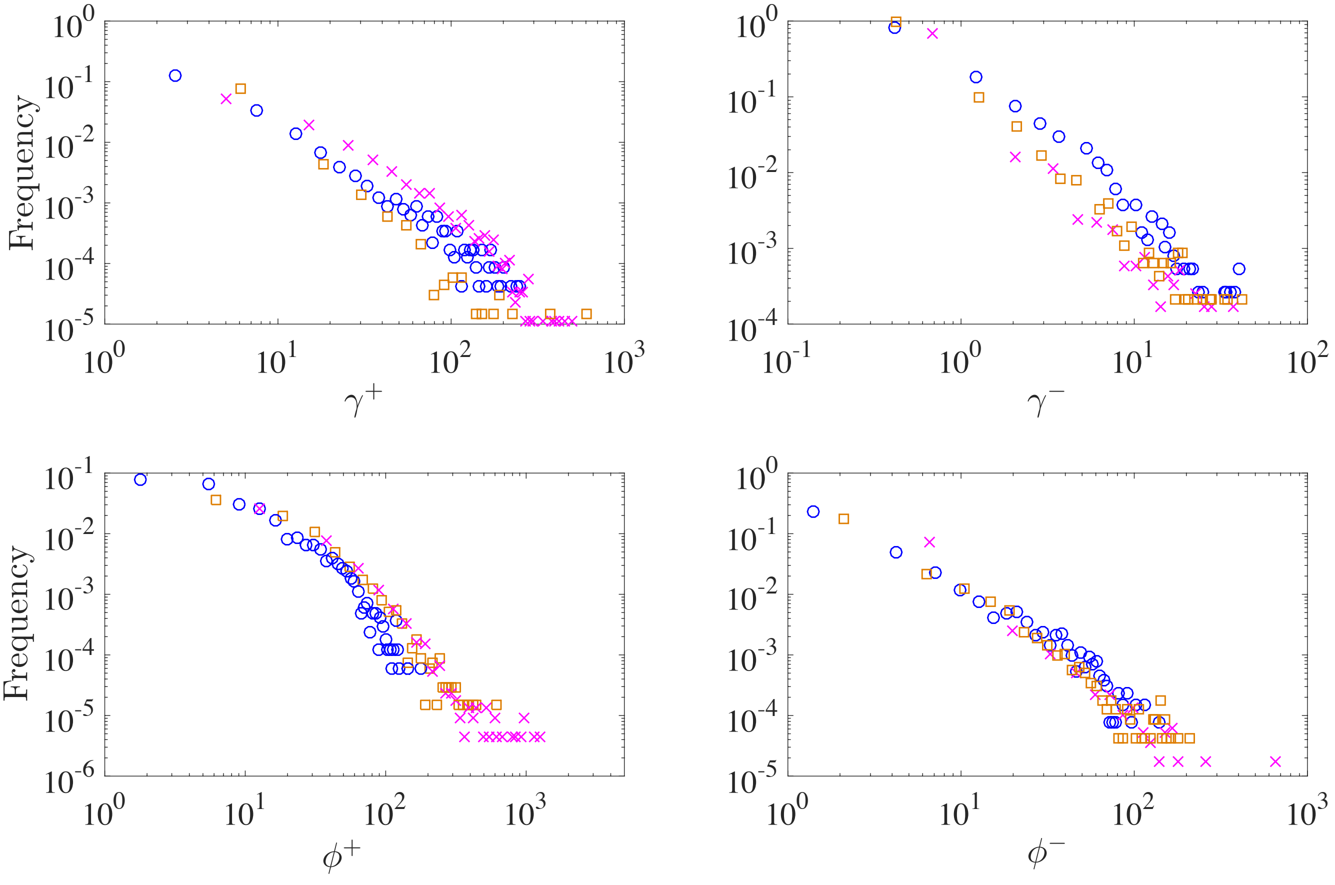

FIG. S.1: **Distributions of the number of positive/negative reciprocated and unreciprocated ratings received by each user**. Empirical distributions of the reciprocated positive ratings γ^+ (top-left panel), reciprocated negative ratings γ^- (top-right panel), unreciprocated positive ratings ϕ^+ (bottom-left panel), unreciprocated negative ratings ϕ^- (bottom-right panel) received by each user. Such quantities correspond to the ones introduced in Eqs. (1) and (2) of the main paper. In all plots blue circles represent Slashdot data, pink crosses represent Epinions data, and orange squares represent Wikipedia data.

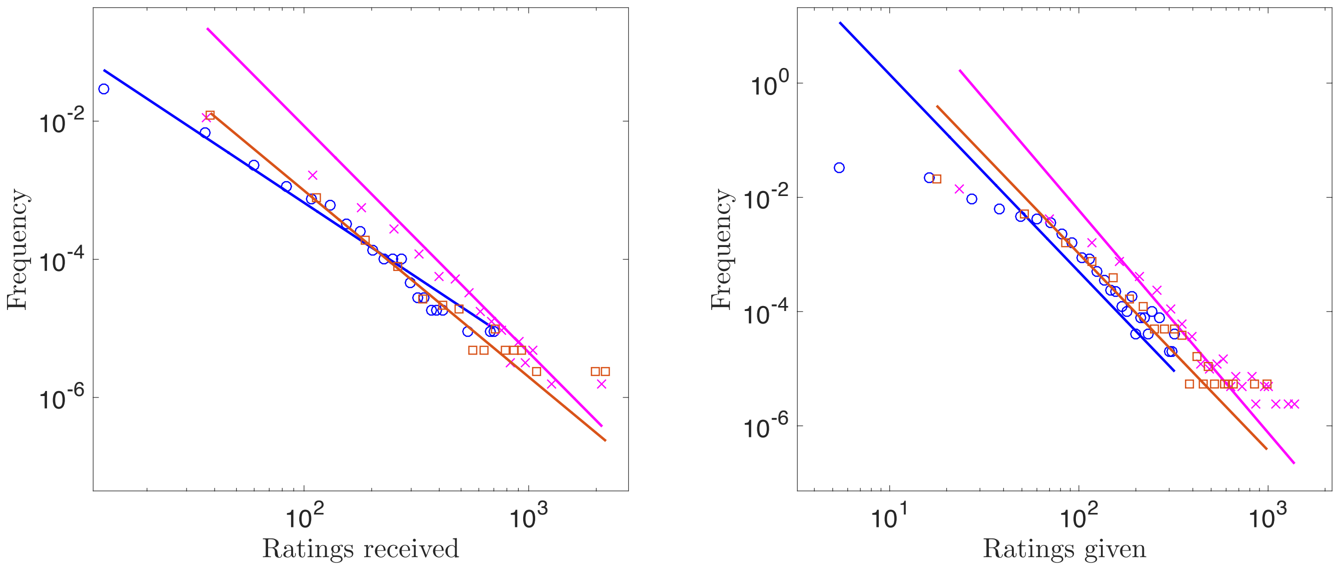

FIG. S.2: **Distribution of the ratings received and given by each user**. Empirical distribution of the ratings received (left panel) and given (right panel) by each node, plotted in log-log scale. Blue circles represent Slashdot data, pink crosses represent Epinions data, and orange squares represent Wikipedia data. Solid lines (of the same colours) show power law of the form $p(x) \sim x^{-\alpha}$ calibrated on the data with the method introduced in [2]. For the distribution of received ratings we find $\alpha = 2.16$ (Slashdot), $\alpha = 3.28$ (Epinions), and $\alpha = 2.70$ (Wikipedia). In the case of given ratings we find $\alpha = 3.45$ (Slashdot), $\alpha = 3.89$ (Epinions), and $\alpha = 3.45$ (Wikipedia).

In Fig. S.3 we report the empirical probability densities and the cumulative distributions of the reputations, computed as per Eq. (3) of the main paper, of all users in the platforms we analyse. As a straightforward consequence of the marked prevalence of positive ratings (see Table I of the main paper), a substantial fraction of users have reputations in the upper end of the spectrum. In particular, 35.9% of Slashdot users, 58.1% of Epinions users, and 45.1% of Wikipedia have an "immaculate" reputation $R = 1$. This is clearly visible from the jump at $R = 1$ in the right panel in Fig. S.3.

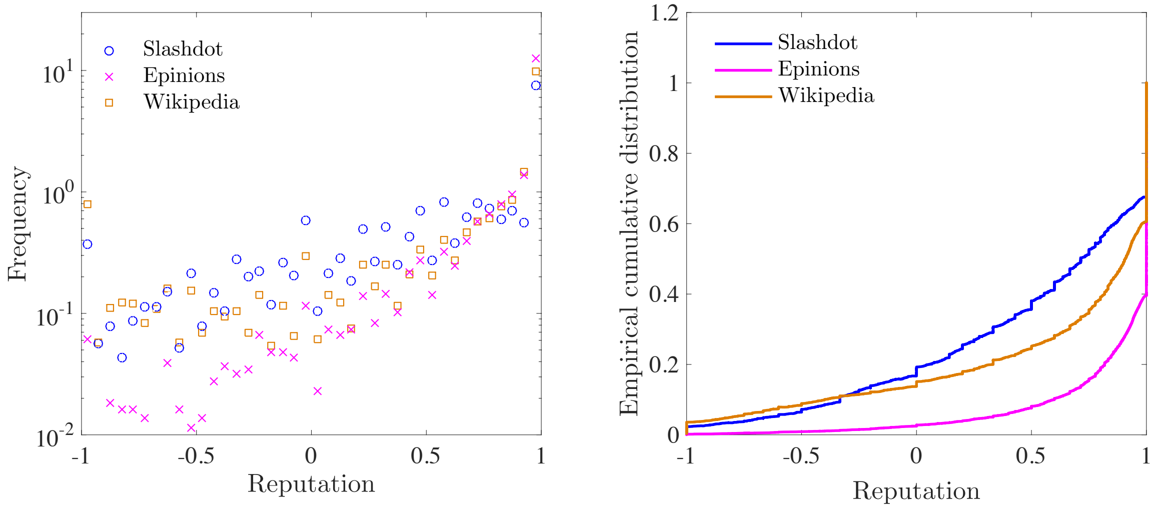

FIG. S.3: **Distributions of reputations.** The left panel shows the empirical probability density functions of the reputations in the three platforms we analyze, while the right panel shows the corresponding cumulative distributions. Reputations are computed as per Eq. (3) of the main paper.

S.2. CONVERGENCE OF REWIRING PROCEDURE

As discussed in the main paper, the rewiring operations we carry out in order to sample configurations from our null model ensembles are repeated until a steady state is reached, which in the long run is ensured by the probabilistic rule in Eq. (5), where β plays the role of an inverse temperature in a physical system. In Fig. S.4 we provide evidence that this is indeed the case by showing the autocorrelation function of the positive reciprocity measured in Slashdot as the rewiring procedure of null model 1 is carried out.

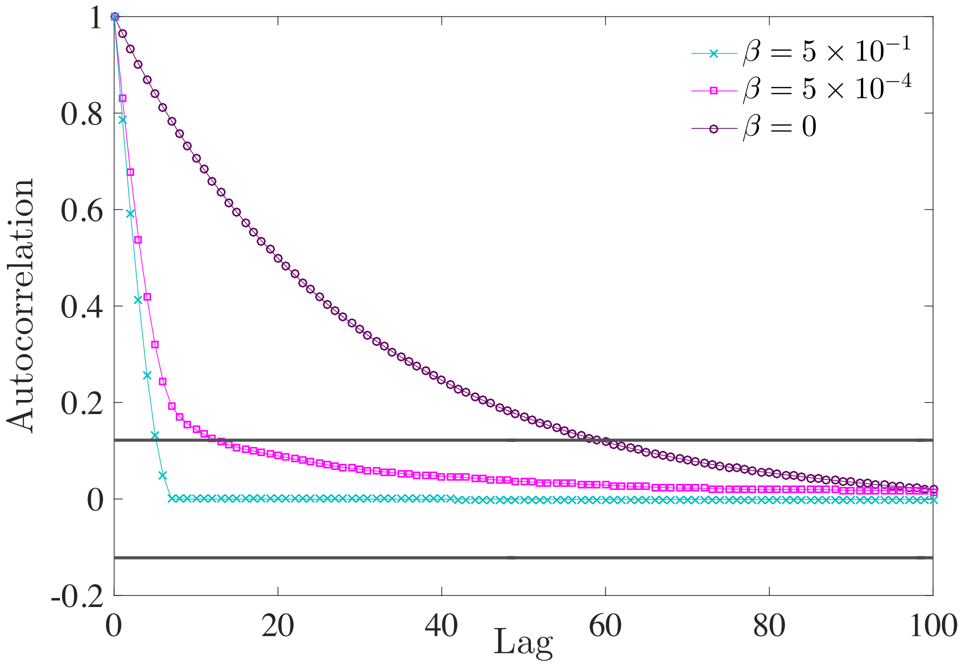

FIG. S.4: **Convergence of the rewiring procedure to a stationary state.** Circles, squares and crosses denote the autocorrelation function of the positive reciprocity in Slashdot as measured during the rewiring procedure described in the main paper. Different curves refer to different values of β (shown in the legend), and in all cases the positive reciprocity target τ^+ is set to 80% of the positive reciprocity ρ^+ measured in the empirical network. Each time lag represents 10^3 attempted rewiring moves. The solid horizontal lines denote the 95% confidence level interval obtained under a null hypothesis of no autocorrelation. As it can be seen, in each case the rewiring procedure reaches a stationary state, albeit with different speeds. Indeed, convergence in the completely randomized case ($\beta = 0$) is much slower than in more "selective" cases characterized by higher values of β.

S.3. GENERAL RESULTS FOR REPUTATION IN NULL MODEL 1

Fig. S.5 shows the contributions to reputation obtained by averaging over large samples of the first null model introduced in the main paper (NM1) as functions of both the intensity of choice parameter β and the reciprocity target τ^+. As it can be seen, we systematically find that the average contribution to reputation from unreciprocated positive links λ^+_Φ is under-expressed with respect to any null hypothesis, and, symmetrically, we find that the contribution from reciprocated positive links λ^+_Γ is over-expressed. As in Fig. 2 of the main paper, λ^+_Φ has a non monotonic behaviour as a function of the reciprocity target, and it reaches a maximum whose value depends on β, whereas λ^+_Γ always displays a monotonically non-decreasing behaviour both as a function of β and τ^+.

Fig. S.6 shows the dependence of the average contributions to reputation from negative ratings. In analogy with the previous case, the contribution from unreciprocated links is systematically under-expressed while that from reciprocated links is systematically over-expressed. In this case, however, Slashdot displays a behaviour which markedly differs from the one observed in Epinions and Wikipedia. In fact, in the latter networks both λ^-_Φ and λ^-_Γ are monotonically increasing functions of τ^-, which shows that increasing the negative reciprocity target increases both retaliation and constructive negative feedback. Conversely, λ^-_Φ in Slashdot decreases as a function of the target τ^-, i.e. the negative contribution to reputation from constructive feedback decreases as reciprocity is increased. This is suggestive of

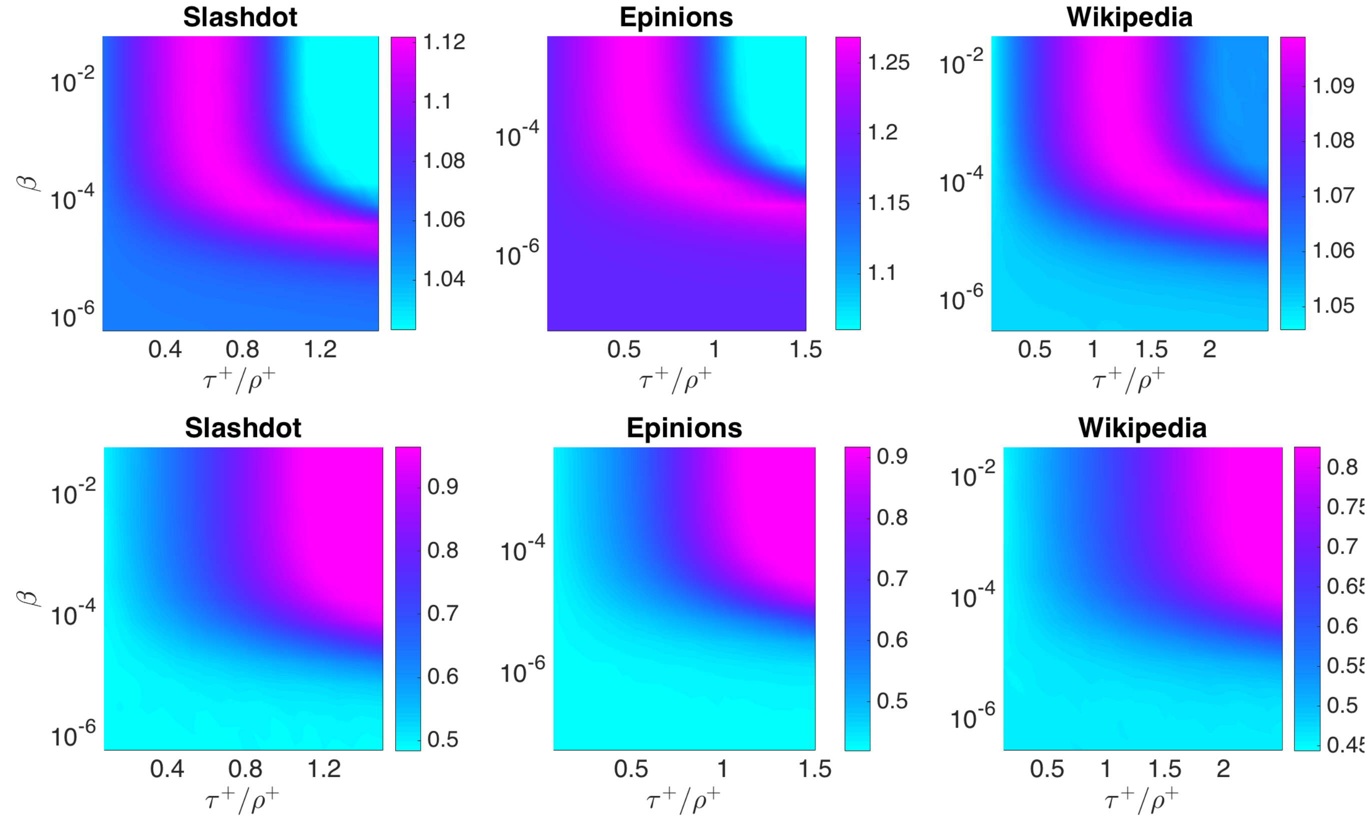

FIG. S.5: **Positive reciprocity bias**. The upper panels show the ratio between the average contribution to reputation from unreciprocated positive ratings λ_{Φ}^{+} measured under our null assumption of random link rewiring, and its value in the empirical networks. Lower panels show the ratio between the average contribution to reputation from reciprocated positive ratings λ_{Γ}^{+} measured under the null assumption and its value in the empirical networks. In each plot such ratio is shown as a function of the intensity of choice parameter β and the reciprocity target τ^+ (normalised by the positive reciprocity ρ^+ of the empirical networks). As it can be seen, in the empirical networks the contribution to reputation from unreciprocated (reciprocated) positive links is systematically under-expressed (over-expressed) with respect to the null hypothesis.

a different signature of genuinely hostile negative interactions, and suggests that reciprocity should be systematically discouraged in polarised environments.

S.4. STATISTICAL PROPERTIES OF PREFERENCE SIMILARITY IN NULL MODEL 2

In the main paper we distinguish between two null models in order to assess the statistical significance of our results compared to a null hypothesis that does not preserve a proxy of homophily in the network, as opposed to a null hypothesis that does. We label this latter as null model 2 (NM2), and the quantity it preserves (together with the reputation of each individual user) is the preference similarity between two nodes, which for a pair of nodes (i, j) reads $S_{ij} = \sum_{\ell=1}^{N} A_{i\ell}A_{j\ell}$. Such a null assumption enforces an additional constraint on the rewiring procedure we employ to build and sample our null models, which is specifically designed to incorporate the empirically observed level of agreement between pairs or users in the platform into our null models.

The rationale behind the above assumption is that users who reciprocate can be reasonably expected to agree more in their approval/disapproval of other peers with respect to users who do not reciprocate. In order to test whether this is indeed the case we compute the preference similarity between pairs of nodes that share a positive reciprocated relationship (i.e. pairs (i, j) such that $A_{ij} = A_{ji} = +1$), and compare its statistical properties to the preference similarity measured between pairs of nodes who share a positive unreciprocated relationship (i.e. pairs (i, j) such that $A_{ij} = +1$ and $A_{ji} = 0$). We label the former quantity as $S^{\leftrightarrow}$ and the latter as $S^{\rightarrow}$. In Tables S.1 and S.2 we report the mean, variance, skewness and kurtosis of those two quantities as computed over all pertinent pairs of nodes in the three platforms we analyse.

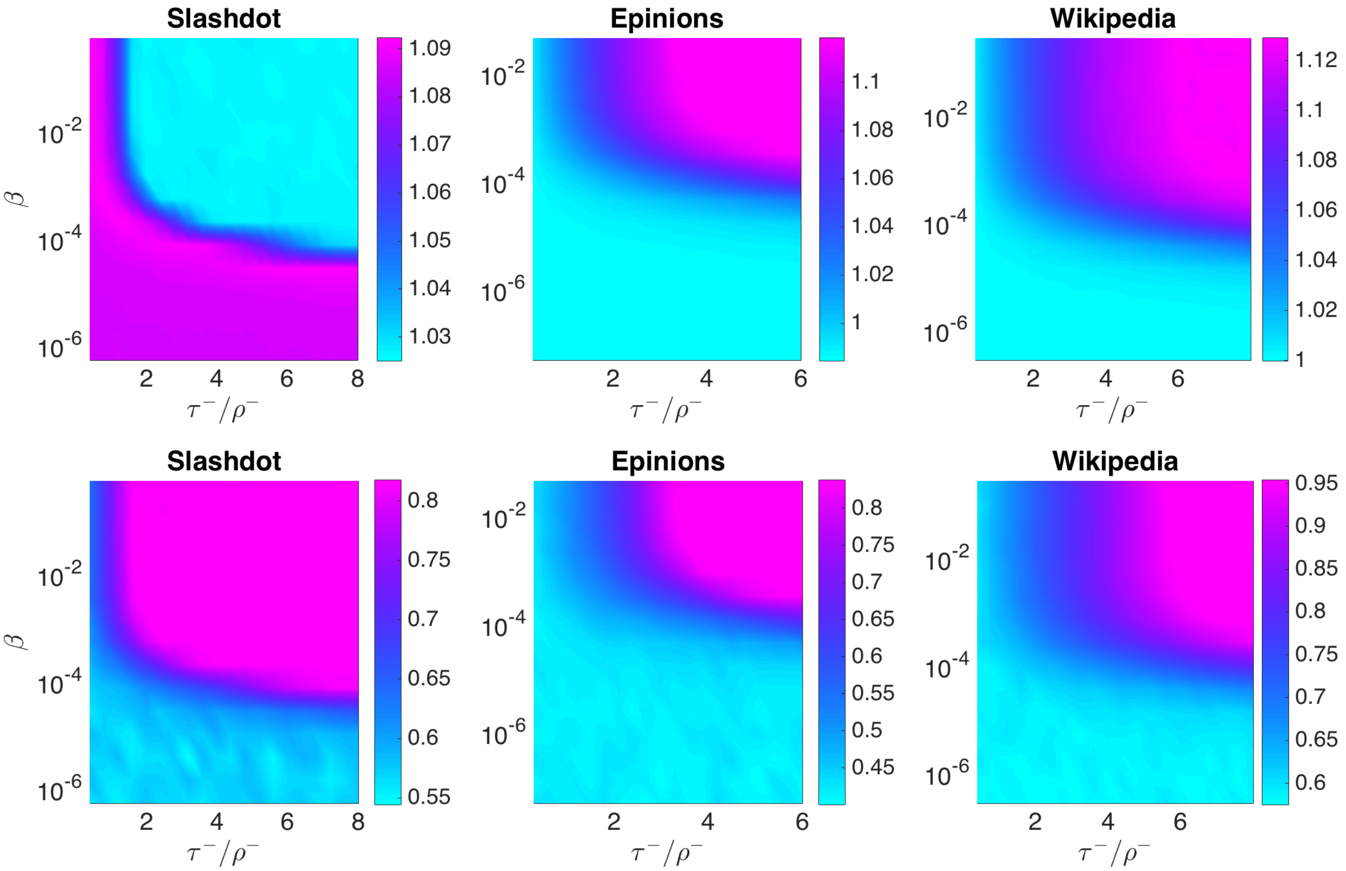

FIG. S.6: **Negative reciprocity bias**. The upper panels show the ratio between the average contribution to reputation from unreciprocated negative ratings λ_Φ^- measured under our null assumption of random link rewiring and its value in the empirical networks. Lower panels show the ratio between the average contribution to reputation from reciprocated negative ratings λ_Γ^- measured under the null assumption and its value in the empirical networks. In each plot such ratio is shown as a function of the intensity of choice parameter β and the reciprocity target τ^- (normalised by the negative reciprocity ρ^- of the empirical networks). As it can be seen, in the empirical networks the contribution to reputation from unreciprocated (reciprocated) negative links is systematically under-expressed (over-expressed) with respect to the null hypothesis.

As it can be seen, in all three networks $S^{\leftrightarrow}$ has a markedly larger mean than $S^{\rightarrow}$, which signals that indeed, on average, users who reciprocate tend to agree more than users who do not. However, both the distributions of $S^{\leftrightarrow}$ and $S^{\rightarrow}$ are significantly leptokurtic and skewed to the right, due to the presence of very active pairs of users with strong preference similarity. Interestingly, this behaviour is much more pronounced in the distributions of $S^{\rightarrow}$, meaning that the largest outliers in preference similarity are observed between pairs of nodes who do not reciprocate.

As an additional robustness check, we test whether the distributions of $S^{\leftrightarrow}$ and $S^{\rightarrow}$ are compatible with those observed under NM2 (at positive reciprocity targets kept equal to the networks' empirical reciprocity, i.e. $\tau^+ = \rho^+$). In Fig. S.7 we plot visual comparisons of the empirical distributions of such quantities and the ones obtained by sampling configurations of NM2, and in Tables S.1 and S.2 we compare the empirical moments with the corresponding 99% significance level intervals obtained with NM2 (we mark with an asterisk the empirical moments that are compatible with such intervals).

As it can be seen, NM2 generally underestimates the empirical means, but still preserves the empirically observed fact that the mean in the distribution of $S^{\leftrightarrow}$ is significantly larger than the one of $S^{\rightarrow}$ (indeed, in all three networks the 99% confidence level intervals for the two means are mutually incompatible). Analogously, with the exception of $S^{\leftrightarrow}$ in Wikipedia, NM2 systematically underestimates the empirical variances.

Higher order moments present a more heterogeneous picture. In fact, NM2 preserves the distributional properties of Slashdot in very good detail, as both the skewness and kurtosis of $S^{\leftrightarrow}$ and $S^{\rightarrow}$ are compatible with the empirically measured ones. On the other hand, NM2 captures well the distributional properties of $S^{\leftrightarrow}$ in Epinions while overestimating the skewness and kurtosis of the corresponding quantity in Wikipedia. Symmetrically, it captures well the properties of $S^{\rightarrow}$ in Wikipedia while underestimating the skewness and kurtosis in Epinions.

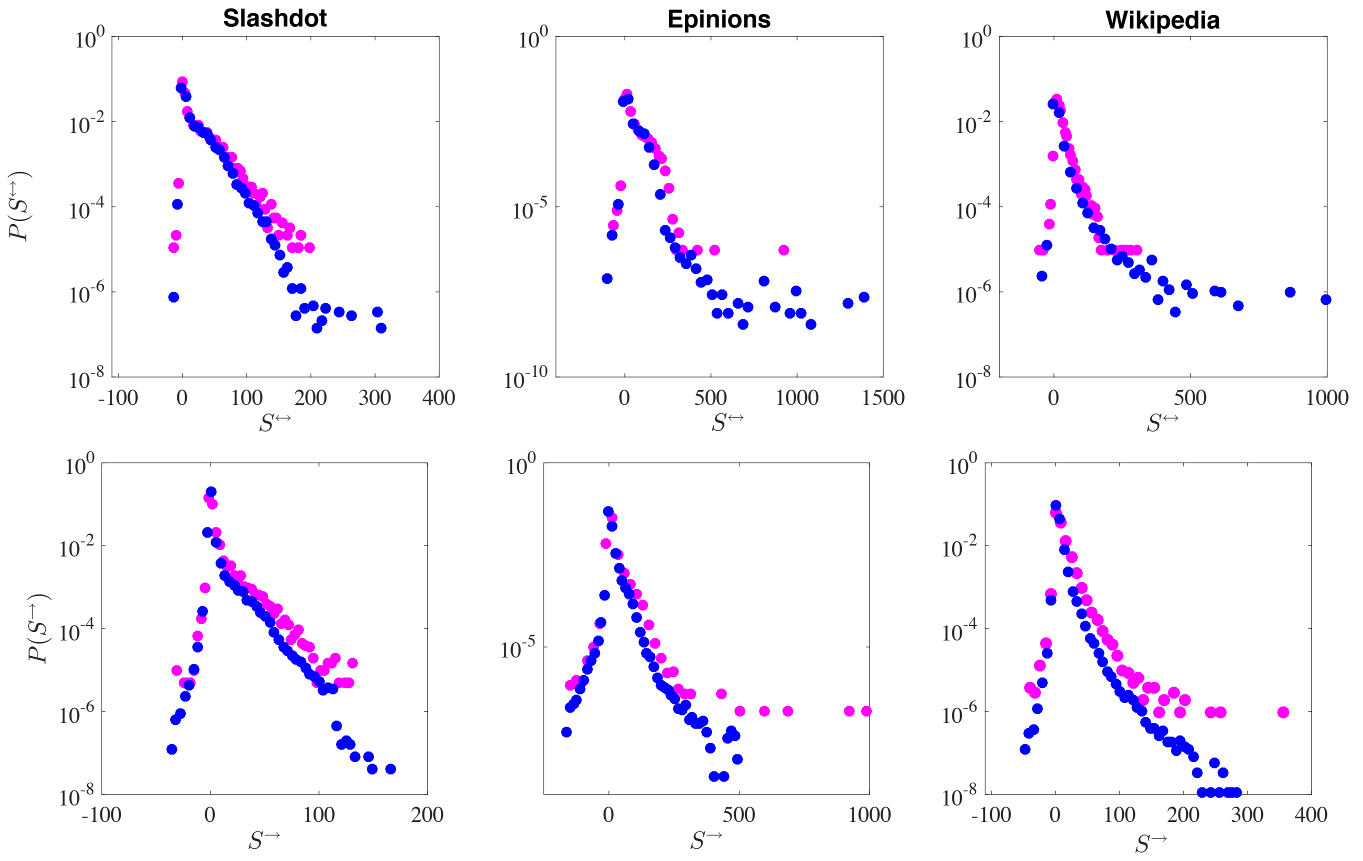

FIG. S.7: **Distribution of preference similarity.** Distributions of the preference similarity $S^{\leftrightarrow}$ between pairs of nodes that share a positive reciprocated relationship (top panels) and of the preference similarity $S^{\rightarrow}$ between pairs of nodes that share one unreciprocated relationship. In all plots blue dots refer to the empirical networks, while pink dots refer to results obtained by averaging over NM2.

	Empirical networks				Null model 2			
	Mean	Variance	Skewness	Kurtosis	Mean	Variance	Skewness	Kurtosis
Slashdot	14.2	4.84×10^2	2.26^*	9.17^*	$[11.5; 11.7]$	$[3.31; 3.44] \times 10^2$	$[2.22; 2.41]$	$[8.73; 11.86]$
Epinions	31.0	1.95×10^3	2.36^*	10.1^*	$[23.2; 23.5]$	$[1.13; 1.16] \times 10^3$	$[2.18; 2.88]$	$[7.7; 37.7]$
Wikipedia	20.2	$3.89 \times 10^{2*}$	3.07	22.0	$[10.8; 11.4]$	$[2.86; 4.89] \times 10^2$	$[6.6; 16.0]$	$[0.89; 5.36] \times 10^2$

TABLE S.1: **Statistical properties of $S^{\leftrightarrow}$.** Moments of the distribution of $S^{\leftrightarrow}$ across all pairs of nodes that share a positive reciprocated relationship in the empirical networks, and 99% confidence level intervals of the same moments computed in NM2 (for $\tau^+ = \rho^+$). Empirical moments marked with an asterisk are those that are compatible with the corresponding confidence level interval in the null models.

	Empirical networks				Null model 2			
	Mean	Variance	Skewness	Kurtosis	Mean	Variance	Skewness	Kurtosis
Slashdot	3.11	72.2	5.11^*	38.2^*	$[0.42; 3.04]$	$[3.0; 63.1]$	$[4.72; 11.42]$	$[0.33; 2.67] \times 10^2$
Epinions	11.30	3.64×10^2	4.88	87.4	$[4.68; 7.52]$	$[0.74; 2.48] \times 10^2$	$[3.19; 4.86]$	$[24.7; 55.8]$
Wikipedia	7.15	94.4	3.93^*	46.4^*	$[1.03; 5.80]$	$[3.2; 60.6]$	$[2.39; 6.21]$	$[13.4; 79.7]$

TABLE S.2: **Statistical properties of $S^{\rightarrow}$.** Moments of the distribution of $S^{\rightarrow}$ across all pairs of nodes that have share an unreciprocated positive relationship in the empirical networks, and 99% confidence level intervals of the same moments computed in NM2 (for $\tau^+ = \rho^+$). Empirical moments marked with an asterisk are those that are compatible with the corresponding confidence level interval in the null models.

All in all, it can be concluded that NM2 succeeds at preserving some non-trivial trends and properties observed in the empirical networks, while also preserving at least the correct order of magnitudes of the quantities it does not fully capture in a statistical sense.

S.5. A NULL MODEL BASED ON LINK AND SIGN RESHUFFLING

As a further check of the robustness of our results, we test the statistical significance of the features observed in the empirical networks against a null hypothesis based on random link and sign reshuffling. Namely, we perform the same rewiring operations carried out for null model 1 in the main paper (top-left picture in Figure 1 of the main paper) on the unsigned networks where each entry is taken equal to $|A_{ij}|$, and later randomly reassign $+/-$ signs on the links in the same proportions as they occur in the empirical networks. This procedure randomises the network topology while preserving its overall heterogeneity in terms of the unsigned degree of each node $k_i = \phi^+ + \phi^- + \gamma^+ + \gamma^-$ (see Eqs. (1) and (2) in the main paper), which corresponds to the overall number of ratings received by each user. By reassigning signs at the end of the random rewiring procedure we therefore build network configurations where all correlations between positive/negative links are removed (except for those due to the higher density of positive links).

	Empirical networks					
	ρ^+	ρ^-	λ^+_Φ	λ^+_Γ	λ^-_Φ	λ^-_Γ
Slashdot	41.3%	15.9%	0.0323	0.0355	0.0340	0.0522
Epinions	42.4%	7.70%	0.0156	0.0220	0.0236	0.0157
Wikipedia	17.6%	8.50%	0.0270	0.0325	0.0362	0.0321
	Null model					
Slashdot	$[1.58; 1.82]\%$	$[0.32; 0.66]\%$	$[0.0343; 0.0347]$	$[0.0176; 0.0207]$	$[0.0336; 0.0350]$	$[0.0148; 0.0286]$
Epinions	$[2.58; 2.75]\%$	$[0.17; 0.34]\%$	$[0.0189; 0.0190]$	$[0.0085; 0.0091]$	$[0.0184; 0.0191]$	$[0.0069; 0.0105]$
Wikipedia	$[2.77; 3.05]\%$	$[0.36; 0.66]\%$	$[0.0295; 0.0297]$	$[0.0139; 0.0155]$	$[0.0286; 0.0297]$	$[0.0099; 0.0197]$

TABLE S.3: **Results from a null model based on link and sign reshuffling.** In the first four rows we report, for the sake of convenience, the values of positive and negative reciprocity $\rho^\pm$ and the average contributions to reputation from reciprocated ($\lambda^\pm_\Gamma$) and unreciprocated ($\lambda^\pm_\Phi$) links measured in the empirical networks, while in the next four rows we report the 99% confidence level intervals for the corresponding quantities measured under a null assumption of random link and sign reshuffling.

Table S.3 summarises our findings in such null model. Namely, we report the values of positive/negative reciprocity $\rho^\pm$ and the contributions to reputation from reciprocated ($\lambda^\pm_\Gamma$) and unreciprocated ($\lambda^\pm_\Phi$) links as measured in the empirical networks (whose values are also reported in Tables 1, 2, and 3 of the main paper) and in the aforementioned null model. From a qualitative point of view, in most cases we observe the same results we obtained when analysing the results from the two null models discussed in the main paper. Namely:

- In the empirical networks both positive and negative reciprocity are markedly over-expressed (i.e. by more than one order of magnitude) with respect to the null assumption.
- The contribution to reputation from positive unreciprocated (reciprocated) activity λ^+_Φ (λ^+_Γ) in the empirical networks is under-expressed (over-expressed) with respect to the null assumption.
- Whereas in all three networks we analyse we have $\lambda^+_\Gamma > \lambda^+_\Phi$, i.e. on average one reciprocated link contributes to reputation more than one unreciprocated link, under the above null assumption we observe the opposite relationship.
- The average contribution to reputation from reciprocated negative links (λ^-_Γ) in the empirical networks is systematically over-expressed with respect to the above null assumption.

Altogether, the above points confirm that the reciprocity bias persists against a null assumption of random link and sign reshuffling in the positive case. On the other hand, it should be noted that the contribution to reputation from unreciprocated negative links (λ^-_Φ) measured in the empirical networks is compatible with the one measured under the above null assumption in the case of Slashdot, and over-expressed in the case of Epinions and Wikipedia. This is at odds with the observations made in NM1 (see Section S.3), and indicates that a random redistribution of the ratings either preserves or underestimates the contribution to reputation from unilateral negative feedback.

S.6. LINK ELIMINATION

One of our main results concerns the fragility of the states observed in real-life P2P networks. Indeed, as we show in the main paper, the random elimination of a small fraction (i.e. $3-11\%$ depending on the network) of

reciprocated positive links is sufficient to remove the reciprocity bias and make the contribution to reputation from positive unreciprocated ratings (λ_Φ^+) and reciprocated ratings (λ_Γ^+) statistically compatible. Let us remark that the protocol chosen to carry out the rating elimination procedure is crucially important to keep the fraction of removed ratings so low. Indeed, the above efficiency is achieved only when selecting *pairs of users* at random. Eliminating reciprocated ratings at random requires instead the elimination of a much larger number of ratings, as it can be seen in Fig. S.8. Given the heavy tailed nature of the distributions of ratings given and received by each node (see Fig. (S.2)), this means that the most efficient way to decrease the contribution to reputation from reciprocated activity is to eliminate the reciprocated ratings between users with a lower number of ratings. In contrast, removing any rating with equal probability amounts to preferentially removing ratings between high activity users, i.e. hubs in the P2P network. We discuss in the main paper how this result is meaningful from the viewpoint of user incentives.

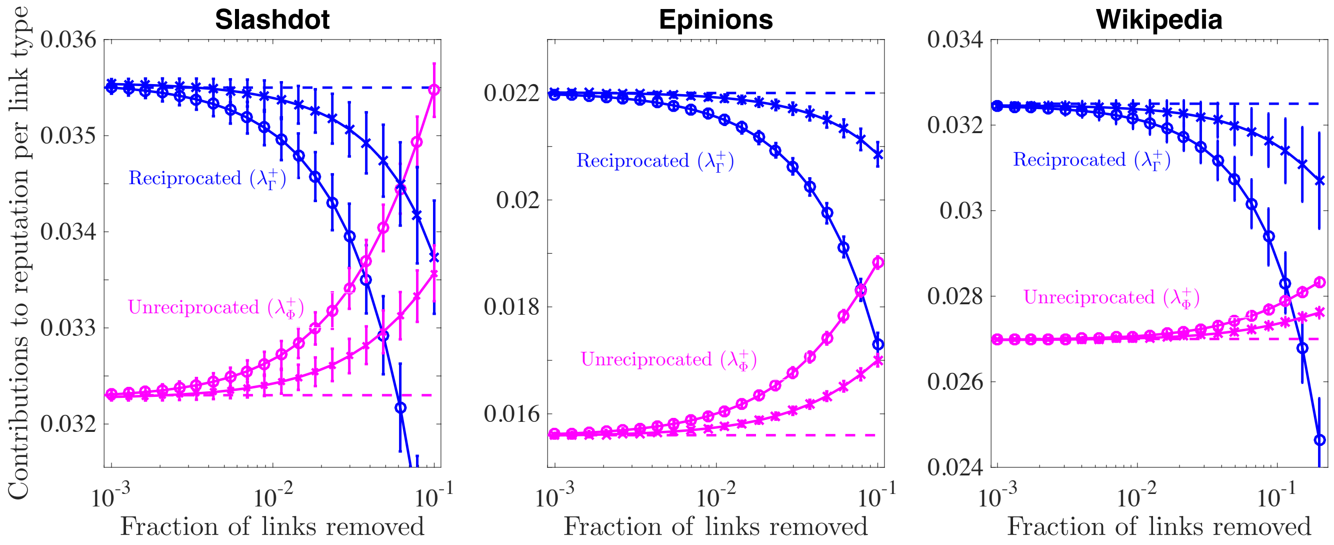

FIG. S.8: **Comparison between the performance of different link elimination protocols in removing the reciprocity bias.** Solid lines show the average contribution to reputation from unreciprocated (λ_Φ^+, pink) and reciprocated (λ_Γ^+, blue) positive links as a function of the fraction of reciprocated positive links removed from the network. Circles represent the behaviour of such quantities when a random node selection protocol is followed, i.e. nodes are chosen at random with uniform probability and reciprocated positive links between them, if any, are removed (this case corresponds to the one shown in Fig. 3 of the main paper). Crosses refer instead to a random link selection protocol, where links are removed with uniform probability. In the former case the majority of links removed are between low degree nodes, whereas in the latter case the elimination procedure targets hubs with higher probability. The dashed lines represent the values of λ_Γ^+ (upper line) and λ_Φ^+ (lower line) in the original networks. Error bars represent 99% confidence level intervals.

S.7. ROBUSTNESS WITH RESPECT TO DIFFERENT THRESHOLDS

All the results in our paper have been obtained by restricting empirical networks to a high participation core of actively engaged users with at least $t = 10$ ratings (either given or received). In this Section we provide evidence that all the main results we discuss in the main paper are robust with respect to changes in the threshold t. In particular, we discuss the case of an *extended participation core* to show the effects of a lower threshold ($t = 5$), which allows to take into account the contribution of less actively engaged users. In Table S.4 we report the number of nodes and positive/negative links for such networks. As it can be seen, consistently with the case $t = 10$, we still observe a large prevalence of positive links, which represent 80% or more of the total links. The only slight qualitative difference with respect to the networks used in the main papers is an increase in sparsity, which is due to the inclusion of a substantial number of nodes with low activity.

In Tables S.5 and S.6 we summarise the values of positive/negative reciprocity $\rho^\pm$ we measure in the participation cores for $t = 5$, as well as the corresponding basal reciprocity $\rho_0^\pm$ and saturation reciprocity $\rho_{\mathrm{SAT}}^\pm$ we obtain by averaging over the two classes of null models we consider with $\beta = 0$ and $\beta \to \infty$, respectively. By comparing such values in the $t = 10$ and $t = 5$ cases one can notice that essentially the reciprocity levels observed in the empirical networks and their relative magnitude with respect to those computed over null model samples remain qualitatively

	N	L^+	L^-	ξ^+	ξ^-
Slashdot	$8,982$	$159,861$	$47,437$	2.0×10^{-3}	5.9×10^{-4}
Epinions	$14,192$	$496,545$	$52,643$	2.5×10^{-3}	2.6×10^{-4}
Wikipedia	$8,995$	$214,692$	$37,121$	2.7×10^{-3}	4.6×10^{-4}

TABLE S.4: **Network statistics in the extended participation core.** Number of users N, number of positive (L^+) and negative (L^-) ratings, and sparsity $\xi^\pm$ in the participation core obtained for $t = 5$.

		Null model 1		Null model 2	
	ρ^+	ρ_0^+	ρ_{SAT}^+	ρ_0^+	ρ_{SAT}^+
Slashdot	35.4%	[1.23; 1.46]%	[39.8; 40.0]%	[3.08; 3.18]%	[36.2; 36.4]%
Epinions	41.0%	[1.89; 1.99]%	[46.3; 46.4]%	[3.31; 3.35]%	[41.5; 41.6]%
Wikipedia	15.3%	[2.39; 2.55]%	[32.2; 32.5]%	[2.33; 2.39]%	[21.7; 22.0]%

TABLE S.5: **Over-expression of positive reciprocity in the extended participation cores.** Comparison between the positive reciprocity ρ^+ observed in the extended participation cores of the three networks we analyse and the 99% confidence level intervals for the corresponding "basal" levels ρ_0^+ and saturation levels ρ_{SAT}^+ obtained under a null hypothesis of random link rewiring constrained to preserve each user's reputation (null model 1), and a null hypothesis further constrained to also preserve the preference similarity of each pair of nodes (null model 2).

		Null model 1		Null model 2	
	ρ^-	ρ_0^-	ρ_{SAT}^-	ρ_0^-	ρ_{SAT}^-
Slashdot	13.8%	[0.21; 0.40]%	[21.2; 21.8]%	[6.52; 6.89]%	[16.8; 17.2]%
Epinions	6.88%	[0.80; 1.10]%	[22.0; 22.5]%	[5.13; 5.55]%	[16.0; 16.5]%
Wikipedia	7.38%	[1.55; 1.95]%	[45.0; 45.6]%	[6.93; 7.90]%	[30.7; 31.2]%

TABLE S.6: **Over-expression of negative reciprocity in the extended participation cores.** Comparison between the negative reciprocity ρ^- observed in the extended participation cores of the three networks we analyse and the 99% confidence level intervals for the corresponding "basal" levels ρ_0^- and saturation levels ρ_{SAT}^- obtained under a null hypothesis of random link rewiring constrained to preserve each user's reputation (null model 1), and a null hypothesis further constrained to also preserve the preference similarity of each pair of nodes (null model 2).

very similar. As a consequence of this fact, we still observe a substantial over-expression of reciprocity, especially in the positive case, with respect to the basal levels $\rho_0^\pm$, and we still observe that Slashdot and Epinions display positive reciprocity close to their respective saturation levels $\rho_{\mathrm{SAT}}^\pm$ (especially those computed under NM2), whereas Wikipedia's positive and negative reciprocity remain quite far from the corresponding saturation levels.

In full analogy with the results reported in the main paper, we find that in the extended participation cores the average contribution λ_Γ^+ to reputation from positive reciprocated ratings is systematically higher than the average contribution λ_Φ^+ from unreciprocated positive ones. Also in analogy with the restricted participation core analysed in the main paper, we again observe that only in Slashdot the contribution to reputation from reciprocated negative ratings exceeds that from unreciprocated negative ones (i.e. $\lambda_\Gamma^- > \lambda_\Phi^-$).

	λ_Φ^+	λ_Γ^+	λ_Φ^-	λ_Γ^-
Slashdot	0.0391	0.0504	0.0411	0.0632
Epinions	0.0207	0.0311	0.0344	0.0251
Wikipedia	0.0328	0.0397	0.0469	0.0409

TABLE S.7: **Evidence that reciprocated ratings contribute more to reputation than unreciprocated ones in the extended participation core.** Average contribution to reputation from each link category: $\lambda_\Phi^\pm$ denote the average contribution from a positive/negative unreciprocated rating, while $\lambda_\Gamma^\pm$ denote the average contribution from a positive/negative reciprocated rating.

The above consistency of results is also detected in terms of reciprocity bias. In the main paper (i.e. on the $t = 10$ participation core) we observe the following two features:

- A systematic over-expression of the average contribution to reputation from positive/negative reciprocated ratings ($\lambda_\Gamma^\pm$) in the empirical networks with respect to any null hypothesis (i.e. for any value of β or $\tau^\pm$) preserving the networks' reputation landscape and the networks' local preference similarity structure.
- For large values of β (i.e. when the rewiring procedure is made very selective), the average contribution to

reputation from positive unreciprocated ratings in the null models is larger than the average contribution from reciprocated positive ratings (i.e., $\lambda_\Phi^+ > \lambda_\Gamma^+$) over a wide range of reciprocity targets τ^+, as opposed to what is observed in the empirical networks.

Both the above points are illustrated for the $t = 5$ participation core in Fig. S.9, which closely resembles Fig. 2 of the main paper.

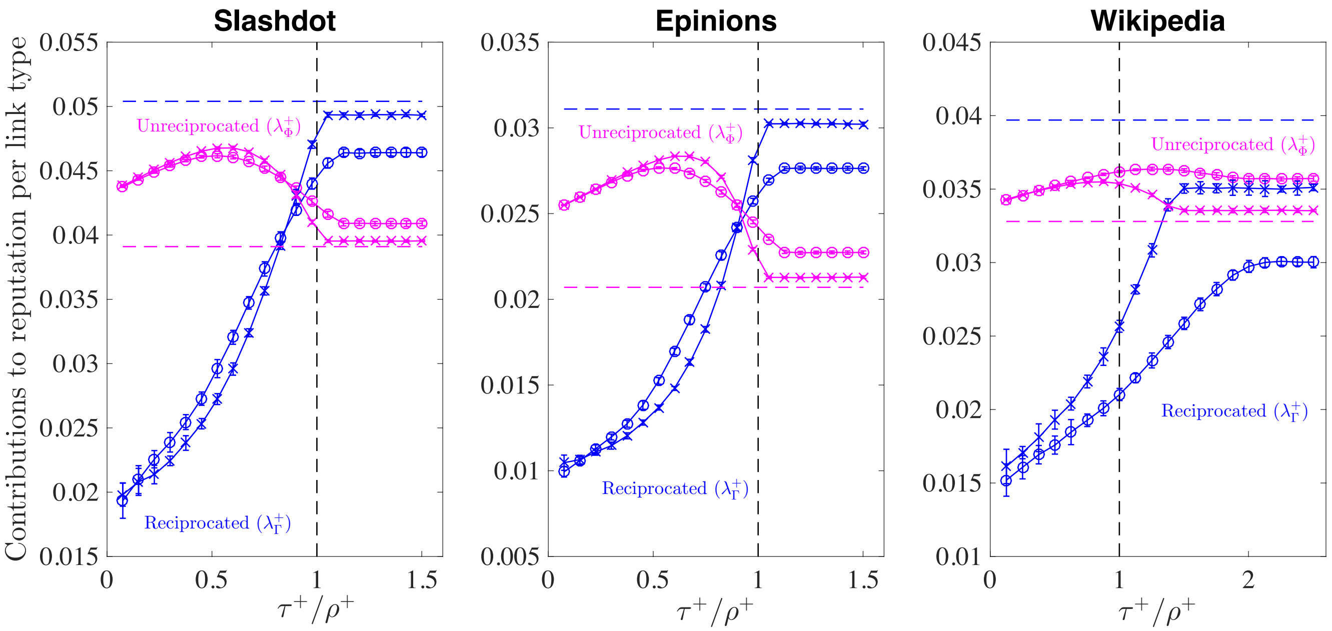

FIG. S.9: **Reciprocity bias in the extended participation core**. Behaviour of the average contribution to reputation from unreciprocated positive ratings (λ_Φ^+, pink) and reciprocated positive ratings (λ_Γ^+, blue) under two null hypotheses of random link rewiring designed to produce a predefined positive reciprocity target ρ^+ in the extended participation core. Circles refer to a null hypothesis constrained to preserve the reputation of each user (null model 1), while crosses refer to a null hypothesis further constrained to also preserve the preference similarity of each pair of nodes (null model 2). The behaviour of λ_Φ^+ and λ_Γ^+ is shown as a function of the ratio between the reciprocity target τ^+ and the positive reciprocity ρ^+ measured in the actual platforms (first column in Table S.5). Error bars correspond to 99% confidence level intervals. Dashed lines correspond to the values of λ_Φ^+ (pink) and λ_Γ^+ (blue) measured in the actual platforms (i.e. to the values reported in columns 1 and 2, respectively, of Table S.7). The fact that the contribution from reciprocated (unreciprocated) activity in the actual platforms is systematically lower (higher) than under our null hypotheses highlights the existence of the reciprocity bias in the extended participation core.

As discussed in the main paper, we find the reciprocity bias to be a peculiar property of real-life P2P platforms. Indeed, small perturbations are enough to make the contributions to reputation from unreciprocated (λ_Φ^+) and reciprocated (λ_Γ^+) positive ratings statistically compatible. We detect the same phenomenon in the extended participation core: As shown in Fig. S.10, the removal of a small fraction of reciprocated positive ratings is enough to first make λ_Φ^+ and λ_Γ^+ statistically compatible, and to eventually make the former the prevalent contribution to reputation, as opposed to what is observed in the empirical networks. In particular, we find that the removal of 8% randomly selected reciprocated positive ratings in Slashdot (which correspond to less than 3% of the overall positive ratings) is enough to make λ_Φ^+ and λ_Γ^+ statistically compatible. The same result is achieved by removing 6% of the reciprocated positive ratings in Epinions (corresponding to 2.5% of the overall positive ratings) and 10% of the reciprocated positive ratings in Wikipedia (i.e. 1.5% of the overall positive ratings).

In full analogy with the case discussed in Section S.6, we find that the link elimination protocol adopted to remove the ratings plays a crucial role in keeping the overall fractions of removed links so low. Indeed, we find that the most efficient protocol is the one based on the random selection of pairs of nodes and the subsequent elimination of possible reciprocated ratings between them. On the other hand, as shown in Fig. S.10 a protocol based on the random selection of links is much less efficient and entails the removal of substantial portions of links in order to

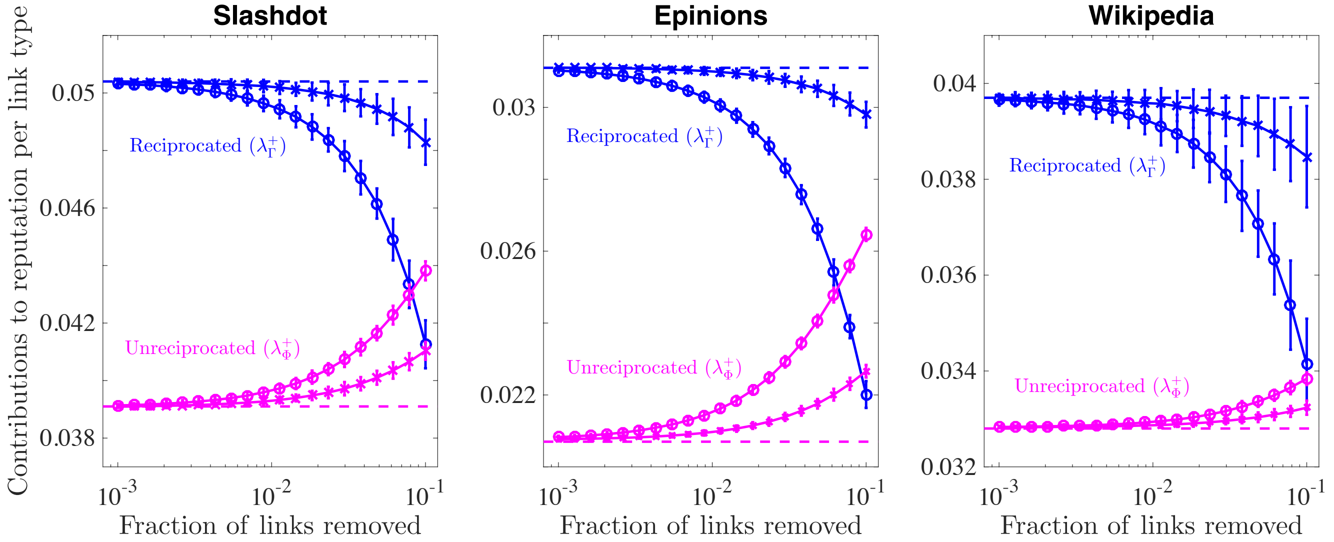

FIG. S.10: **Elimination of the reciprocity bias in the extended participation core**. Solid lines show the average contribution to reputation from unreciprocated (λ^+_Φ, pink) and reciprocated (λ^+_Γ, blue) positive links as a function of the fraction of reciprocated positive links removed from the extended participation core network. Circles represent the behaviour of such quantities when a random node selection protocol is followed, i.e. nodes are chosen at random with uniform probability and reciprocated positive links between them, if any, are removed. Crosses refer instead to a random link selection protocol, where links are removed with uniform probability. In the former case the majority of links removed are between low degree nodes, whereas in the latter case the elimination procedure targets hubs with higher probability. The dashed lines represent the values of λ^+_Γ (upper line) and λ^+_Φ (lower line) in the original networks. Error bars represent 99% confidence level intervals.

achieve statistical compatibility between λ^+_Γ and λ^+_Φ.

---

[1] Caldarelli, G. 2007 *Scale-free networks: Complex webs in nature and technology* Oxford, UK: Oxford University Press.
[2] Clauset, A, Shalizi, CR, Newman, MEJ. 2009 Power-law distributions in empirical data, *SIAM Rev.* **51**:661-703.
